# Supplementary material for: Work Shift, Lifestyle Factors, and Subclinical Atherosclerosis in Spanish Male Workers: A Mediation Analysis
Source: Nutrients. 2021 Mar 26;13(4):1077. doi: 10.3390/nu13041077 (PMC8065668; doi:10.3390/nu13041077)
Supplement: Supplementary file 1 [file nutrients-13-01077-s001.pdf]

Table S1. Food and beverage consumption and nutrient intake of the AWHS participants according to type of work shift.

	**Total** (*n*=2459)	**Central** (*n*=207)	**Rotating M-E** (*n*=1493)	**Rotating M-E-N** (*n*=499)	**Night** (*n*=260)	***p*-value**
**Foods**						
Fruits, g/day	305 ± 181	288 ± 156	303 ± 184	318 ± 184	303 ±174	0.187
Missing, n (%)	*3 (0.1)*	-	*3 (0.2)*	-	-	
Vegetables, g/day	322 ±131	323 ± 114	318 ± 129	338 (±147)	315 ±124	**0.018**
Missing, n (%)	*1 (0.0)*	-	*1 (0.1)*	-	-	
Legumes, g/day	16.2 ± 7.27	15.3 ± 5.90	16.3 ± 7.00	16.5 ± 9.09	15.3 ± 5.63	0.057
Missing, n (%)						
Nuts, g/day	10.4 ± 12.4	10.3 ± 10.8	9.98 ± 12.6	11.4 ± 12.7	10.7 ± 12.1	0.184
Missing, n (%)	*1 (0.0)*	-	-	-	*1 (0.4)*	
Olive oil, g/day	27.6 ± 12.2	27.1 ± 10.9	27.4 ± 12.3	29.6 ± 12.8	25.6 ± 10.3	**<0.001**
Fish, g/day	86.4 ± 46.1	101 ± 46.1	83.8 ± 46.9	91.3 ± 45.6	81.3 ± 38.6	**<0.001**
Processed red meat, g/day	187 ± 71.4	171 ± 62.3	189 ± 73.5	188 ± 69.6	187 ± 68.1	**0.013**
Missing, n (%)	*1 (0.0)*	-	*1 (0.1)*	-	-	
**Nutrients**						
ratio MUFA:SFA	1.62 ± 0.32	1.69 ± 0.31	1.61 ± 0.32	1.65 ± 0.34	1.59 ± 0.29	**<0.001**
Trans fats, g/day	0.86 ± 0.48	0.74 ± 0.42	0.88 ± 0.49	0.87 ± 0.46	0.839 ± 0.47	**<0.001**
Total fiber, g/day	25.4 ± 8.00	24.2 ± 7.66	25.5 ± 8.09	26.1 ± 7.95	24.7 ± 7.73	**0.012**
Sodium, mg/day	3270 ± 1130	2810 ± 999	3360 ± 1140	3270 ± 1140	3170 ± 1050	**<0.001**
Alcohol, g ethanol/day	21.3 ± 20.0	17.9 ± 16.4	21.7 ± 20.4	21.6 ± 19.9	21.6 ± 19.8	0.081
**Beverages**						
Soft drinks, mL/day	64.3 ± 127	38.5 ± 72.8	67.5 ±1 20	61.0 ± 143	72.5 ± 157	**0.012**
Missing, n (%)	*1 (0.0)*	-	*1 (0.1)*	-	-	
Wine, mL/day	92.5 ± 123	77.9 ± 94.3	93.6 ± 126	94.2 ± 123	94.3 ± 123	0.361
Missing, n (%)	*1 (0.0)*	*1 (0.5)*	-	-	-	
Beer, mL/day	299 ± 375	241 ± 290	307 ± 383	302 ± 404	293 ± 331	0.127
Missing, n (%)	*1 (0.0)*	-	*1 (0.1)*	-	-	
Spirits, mL/day	6.90 ± 13.2	6.57 ± 10.6	6.86 ± 13.3	6.66 ± 12.9	7.84 ± 14.4	0.651
Coffee, mL/day	95.8 ± 80.5	101 ± 75.1	92.2 ± 78.7	102 ± 84.8	101 ± 85.4	**0.049**

Data are presented as n (%) or mean ± SD; Central - Central-day shift; M-E – morning/evening rotation shift; M-E-N – morning/evening/night rotation shift; Night – night shift; MUFA – mono-unsaturated fatty acids; SFA – saturated fatty acids, p-value obtained using ANOVA for continuous variables and Chi-square for categorical variables.

Table S2. Estimation of the mediation effect of lifestyle and biological risk factors in the association between work shift (Morning-Evening versus central shift) and the presence of subclinical atherosclerosis

**Mediator**	**Model** [a]	**Natural direct effect** [b]			**Natural indirect effect** [b]			**Total effect** [b]			**% mediated** [b]
		OR	95%CI	*p*-value	OR	95%CI	*p*-value	OR	95%CI	*p*-value	
Lifestyle risks											
Smoking	2	1.19	[0.86; 1.65]	0.296	1.17	[1.08; 1.26]	<0.001	1.39	[1.00; 1.94]	0.052	-
Sleep	2	1.20	[0.85; 1.69]	0.296	1.02	[1.00; 1.04]	0.133	1.22	[0.87; 1.71]	0.250	-
Sitting	2	1.20	[0.85; 1.69]	0.296	0.95	[0.89; 1.02]	0.161	1.14	[0.81; 1.60]	0.442	-
Mediterranean diet	2	1.20	[0.85; 1.69]	0.296	1.04	[0.99; 1.08]	0.082	1.25	[0.89; 1.75]	0.202	-
Coffee	2	1.20	[0.85; 1.69]	0.296	0.98	[0.96; 1.00]	0.121	1.18	[0.84; 1.66]	0.346	-
Metabolic risks									[0.00; 0.00]		
SBP	2	1.13	[0.80; 1.59]	0.495	1.07	[1.02; 1.12]	0.005	1.20	[0.85; 1.70]	0.296	-
Glucose	2	1.23	[0.88; 1.73]	0.232	0.97	[0.95; 0.99]	0.036	1.20	[0.85; 1.69]	0.297	-
HbA1c	2	1.48	[0.93; 2.36]	0.095	1.08	[1.01; 1.16]	0.037	1.60	[1.02; 2.53]	0.043	
BMI	2	1.19	[0.85; 1.68]	0.313	1.01	[0.99; 1.02]	0.473	1.20	[0.85; 1.69]	0.294	-
Waist	2	1.23	[0.87; 1.74]	0.245	1.01	[0.99; 1.03]	0.248	1.24	[0.88; 1.76]	0.221	-
HDL cholesterol	2	1.19	[0.85; 1.67]	0.318	1.01	[0.98; 1.03]	0.508	1.20	[0.85; 1.69]	0.296	-
Triglycerides	2	1.18	[0.84; 1.67]	0.332	1.01	[0.99; 1.03]	0.142	1.20	[0.85; 1.69]	0.296	-
Framingham Risk Score	2	1.16	[0.82; 1.62]	0.339	1.04	[0.99; 1.09]	0.148	1.20	[0.85; 1.69]	0.298	-

Exponentiated coefficient (Odds Ratios (OR) and 95% confidence intervals in brackets). Because of multiple comparisons, Bonferroni-adjusted a p-value below or equal to 0.01 for lifestyle risks, and below or equal to 0.003 for metabolic factors, were considered to be statistically significant. [a] Model 2 adjusted for age, participant's educational level, and lifestyle-related confounding variables, except the lifestyle-related variable under study; Model 3 removing the adjustment for smoking, as they were identified as potential mediator in the pathway of shift work and atherosclerosis [b] Estimations of the natural direct, indirect and total effects for rotating Morning-Evening versus central shift. [c] Complete-case-analysis in 2,429 men for lifestyle-related variables and biological risk factors, except for BMI in 2,423 men, for HbA1c in 1,442 and for waist in 2,375 men.

Table S3. Estimation of the mediation effect of lifestyle and biological risk factors in the association between work shift (night shift versus central shift] and the presence of subclinical atherosclerosis

**Mediator**	**Model** [a]	**Natural direct effect** [b]			**Natural indirect effect** [b]			**Total effect** [b]			**%mediated** [b]
		OR	95%CI	*p*-value	OR	95%CI	*p*-value	OR	95%CI	*p*-value	
Lifestyle risks											
Smoking	2	1.22	[0.81; 1.82]	0.337	1.17	[1.07; 1.28]	0.005	1.42	[0.95; 2.14]	0.090	-
Sleep	2	1.23	[0.81; 1.87]	0.337	1.03	[1.00; 1.07]	0.054	1.27	[0.84; 1.93]	0.260	-
Sitting	2	1.23	[0.81; 1.87]	0.377	0.96	[0.91; 1.02]	0.166	1.18	[0.78; 1.80]	0.431	-
Mediterranean diet	2	1.23	[0.81; 1.87]	0.337	1.03	[0.99; 1.07]	0.096	1.27	[0.83; 1.93]	0.264	-
Coffee	2	1.23	[0.81; 1.87]	0.337	1.00	[0.98; 1.02]	0.983	1.23	[0.81; 1.87]	0.337	-
Metabolic risks											
SBP	2	1.13	[0.74; 1.72]	0.574	1.09	[1.03; 1.16]	0.004	1.23	[0.81; 1.88]	0.335	-
HbA1c	2	1.32	[0.75; 2.32]	0.343	1.07	[1.01; 1.14]	0.027	1.41	[0.80; 2.48]	0.232	
BMI	2	1.21	[0.79; 1.85]	0.376	1.01	[0.98; 1.04]	0.464	1.23	[0.80; 1.87]	0.347	-
Waist	2	1.23	[0.80; 1.90]	0.337	1.04	[1.00; 1.08]	0.075	1.28	[0.83; 1.96]	0.259	-
HDL cholesterol	2	1.24	[0.82; 1.88]	0.311	0.99	[0.96; 1.02]	0.573	1.23	[0.81; 1.87]	0.336	-
Triglycerides	2	1.21	[0.80; 1.84]	0.370	1.02	[0.99; 1.04]	0.254	1.23	[0.81; 1.87]	0.336	-
Framingham Risk Score	2	1.17	[0.77; 1.77]	0.466	1.06	[1.00; 1.13]	0.116	1.23	[0.81; 1.87]	0.338	-

Exponentiated coefficient (Odds Ratios (OR) and 95% confidence intervals in brackets). Because of multiple comparisons, Bonferroni-adjusted a p-value below or equal to 0.01 for lifestyle risks, and below or equal to 0.003 for metabolic factors, were considered to be statistically significant. [a] Model 2 adjusted for age, participant's educational level, and lifestyle-related confounding variables, except the lifestyle-related variable under study; Model 3 removing the adjustment for smoking, as they were identified as potential mediator in the pathway of shift work and atherosclerosis. b Estimations of the natural direct, indirect and total effects for night shift versus central shift. c Complete-case-analysis in 2,429 men for lifestyle-related variables and biological risk factors, , except for BMI in 2,423 men, for HbA1c in 1,442 and for waist in 2,375 men.
